# Draft Genome Sequence of the Moderately Halophilic Bacterium *Pseudoalteromonas ruthenica* Strain CP76

**DOI:** 10.1128/genomeA.00268-13

**Published:** 2013-05-23

**Authors:** Rafael R. de la Haba, Cristina Sánchez-Porro, María José León, R. Thane Papke, Antonio Ventosa

**Affiliations:** Department of Microbiology and Parasitology, Faculty of Pharmacy, University of Sevilla, Sevilla, Spaina; Department of Molecular and Cell Biology, University of Connecticut, Storrs, Connecticut, USAb

## Abstract

*Pseudoalteromonas ruthenica* strain CP76, isolated from a saltern in Spain, is a moderately halophilic bacterium belonging to the *Gammaproteobacteria*. Here we report the draft genome sequence, which consists of a 4.0-Mb chromosome, of this strain, which is able to produce the extracellular enzyme haloprotease CPI.

## GENOME ANNOUNCEMENT

*Pseudoalteromonas ruthenica* is a gammaproteobacterium that was originally isolated from marine invertebrates (mussels and scallops) and was taxonomically characterized by Ivanova et al. (1). Notable features are its extracellular hydrolytic activity (1) and exopolysaccharide production (2), which are involved in biofilm formation (3). Previous research by our group was focused on the isolation and characterization of halophilic bacteria that produce extracellular hydrolytic enzymes useful for biotechnological applications (4, 5, 6). These studies led to the isolation of *Pseudoalteromonas* sp. strain CP76, which was identified as the best producer of an extracellular protease (5). Strain CP76 was isolated from a saltern located in Isla Cristina (Huelva, Spain). This strain is moderately halophilic (7), and the biochemical and molecular features of the protease, designated haloprotease CPI, were studied in detail (5, 8). It has a molecular mass of 38.0 kDa and showed optimal activity at 55°C, pH 8.5 (range, 6 to 10), and 7.5% NaCl (range, 0 to 4 M) (5). These features are highly relevant to biotechnological exploitation. A detailed taxonomic study placed strain CP76 within the species *Pseudoalteromonas ruthenica* (9). The *cpo* gene encoding the extracellular haloprotease CPI was cloned and its nucleotide sequence was analyzed (8). It encodes a 733-residue protein showing sequence similarity to metalloproteases of the M4 family (zincins superfamily). Additional studies showed that the haloprotease CPI is secreted by a type II secretion pathway (8).

A draft genome sequence of *Pseudoalteromonas ruthenica* strain CP76 was obtained using a whole-genome shotgun strategy (10) with Roche 454 pyrosequencing technology on a GS FLX system (Roche Diagnostic, Branford, CT), consisting of single-end reads (430,477 reads, totaling 98,718,018 bp) with approximately 24.6-fold coverage of the entire genome. All reads were assembled into 183 contigs (minimum 100 bp) using Newbler Metrics *de novo* assembler 1.1.03.24 (454 Life Sciences, Branford, CT). Of the contigs, 120 (66%) were a minimum of 570 bp, which were then used to identify open reading frames (ORFs) and functional annotation of these predicted proteins and the rRNA and tRNA genes. The analysis was automated with the Integrative Services for Genomic Analysis (ISGA) (11).

The draft genome is estimated to contain 4,008,122 bp with a G+C content of 47.6% and 3,749 putative ORFs with an average size of 945 bp. The assessed coding density is 88.3%. Furthermore, the strain CP76 contains 3 rRNA operons and a total of 70 tRNA genes.

The genome analysis confirms the presence of *cpo* (coding for haloprotease CPI) and *cpt* (coding for a second protease) genes and genes coding for a type II protein secretion system. In addition, genes for amylase, lipase, and DNase activities were detected.

### Nucleotide sequence accession numbers.

The *Pseudoalteromonas ruthenica* strain CP76 whole-genome shotgun project has been deposited at DDBJ/EMBL/GenBank under the accession number AOPM00000000. The version described in this paper is the first version, AOPM01000000.
